# How Should Forests Be Characterized in Regard to Human Health? Evidence from Existing Literature

**DOI:** 10.3390/ijerph17031027

**Published:** 2020-02-06

**Authors:** Albert Bach Pagès, Josep Peñuelas, Jana Clarà, Joan Llusià, Ferran Campillo i López, Roser Maneja

**Affiliations:** 1 Institute of Environmental Science and Technology (ICTA), Autonomous University of Barcelona (UAB), Z Building, ICTA-ICP, Carrer de les Columnes, UAB Campus, Bellaterra (Cerdanyola del Vallès), 08193 Barcelona, Spain; 2 Environment and Human Health Laboratory (EH2 Lab), Autonomous University of Barcelona (UAB), 08193 Bellaterra, Spain; roser.maneja@uab.cat; 3 CREAF, Campus Universitat Autònoma de Barcelona, Cerdanyola del Vallès, Barcelona, 08193 Catalonia, Spain; josep.penuelas@uab.cat (J.P.); j.llusia@creaf.uab.cat (J.L.); 4 CSIC, Global Ecology Unit CREAF-CSIC-UAB, Bellaterra, Barcelona, 08193 Catalonia, Spain; 5 Geography Department, Autonomous University of Barcelona (UAB), B building, UAB Campus, Bellaterra (Cerdanyola del Vallès), 08193 Barcelona, Spain; jana.13.cb@gmail.com; 6 Garrotxa Pediatric Environmental Health Specialty Unit (PEHSU Garrotxa), Garrotxa Region Pediatric Team, Olot and Garrotxa Region Hospital, Olot, 1780 Catalonia, Spain; FCAMPILLO@hospiolot.cat; 7 Committee on Environmental Health, Spanish Pediatrics Association, 28009 Madrid, Spain; 8 Forest Science and Technology Center of Catalonia, Ctra. de St. Llorenç de Morunys, km 2, 25280 Solsona, Spain

**Keywords:** forest exposure, *shinrin-yoku*, forest characterization, human health, forest management, preventive medicine

## Abstract

The potential of forests as a source of health has been addressed by the scientific community and is now being considered in national forest strategies, management plans and policies. Studies identifying the mechanisms by which forest characteristics may induce these effects on human health are nevertheless scarce. This systematic review of literature on forests and human health with real-life human exposure was conducted to assess the extent to which forests have been studied and described in detail and the extent to which relationships between forest variables and health effects have been reported. The analysis underlines the lack of forest descriptions in 19.35% of the 62 studies selected for review as well as the high heterogeneity of forest variables’ description. Patterns among the articles could not be identified correlating the broader forest variable (forest type) and the most studied health variables identified (blood pressure, pulse rate or/and cortisol levels). These findings, together with previous ex situ researches, suggest the need to ameliorate and incorporate more accurate descriptions of forest variables within human health studies to provide data for forest management and the potential use of these habitats for preventive medicine and clinical practice guidelines.

## 1. Introduction

Interest in the connection between forests and human health is increasing among studies analyzing the effects of nature on human health [1,2,3]. The potential of forests as a source of health has led to numerous studies that provide evidence of the benefits of exposure to these forested ecosystems [1,3,4]. Previous reviews that have compiled the results from these studies report the effects of forests on the following body systems and functions: cardiovascular, respiratory, endocrine, immune, nervous, as well as the impact on mental disorders and psychological well-being [5,6,7,8,9]. Forest exposure has also generally been strongly correlated with stress regulation [9,10]. Although some studies have identified certain elements like biogenic volatile organic compounds (BVOCs) to be potential determinants of the health effects induced by forest exposure [11,12], few have unveiled the mechanisms and pathways by which forests interact with human health [13].

Health promotion is now starting to be integrated into national forest agendas leading changes in strategies, plans and management [1,7,14]. Some studies have highlighted the shift in forest priorities from production and conservation to recreation and promotion of health [14,15]. If health provision is to be integrated into management plans, law and projects, managers, policy-makers and the healthcare community will need data to better understand the specific mechanisms and pathways by which forests’ variables can affect human health.

The aim of this review was thus to analyze the available literature on forests and human health to assess:

- the extent to which forests are studied and described in studies of human health;
- the extent to which patterns can be identified between forest variables and physiological health effects.

## 2. Materials and Methods

Studies published before May 2019 were systematically reviewed following the *Guidelines for Systematic Review and Evidence Synthesis in Environmental Management* [16]. 

### 2.1. Review Scoping

Previous reviews were analyzed to identify relevant terms for configuring a Boolean search: Cho et al. (2017), Hansen et al. (2017), Meyer and Bürger-Arndt (2014), Oh et al. (2017) and Song et al. (2016) [3,6,9,13,17]. The keywords identified were divided into three main blocks: (a) habitat (synonyms of forested environments and terms referring to these areas), (b) activity (action developed in forests that have been tested seeking health outcomes) and (c) health effects (effects of forest exposure on body and health systems or functions). A scoping study was finally conducted using the terms obtained in the review analysis to assess the relevance of each keyword. 

### 2.2. Searches

The keywords identified were combined with Boolean operators considering the three blocks mentioned above to generate the following search: ((forest OR forests OR forested OR woodland* OR jungle OR rainforest) AND (exposure* OR visit* OR bath* OR walk* OR recreation* OR “forest-walking” OR “spending time” OR trip OR “forest-air bathing” OR healing) AND (“human health” OR wellbeing OR well-being OR stress OR health) NOT (biomass OR “forest health” OR “random forest” OR fire OR virus OR “climate change” OR soil OR carbon OR parasite OR radiation OR pathogen)) OR (shinrin-yoku OR shinrinyoku OR “shinrin yoku”).

*Shinrin-yoku* is commonly understood as being in the forest environment and taking in the atmosphere of the forest in expectation of potential curative or therapeutic effects [18]. This term was thus incorporated in the search but was not combined with other keywords as it implies a wide range of activities or actions in a forested environment for health purposes. 

The Web of Science (WOS) and PubMed were selected for the systematic search. Terms were searched in WOS for topic without any other filter, but terms were searched in PubMed for all fields containing the terms and sorted by clinical studies and trials, comparative and observational studies and randomized control trials. The results from PubMed were also filtered by experiments only with humans and articles only written in English. 

### 2.3. Screening

**Figure 1:**
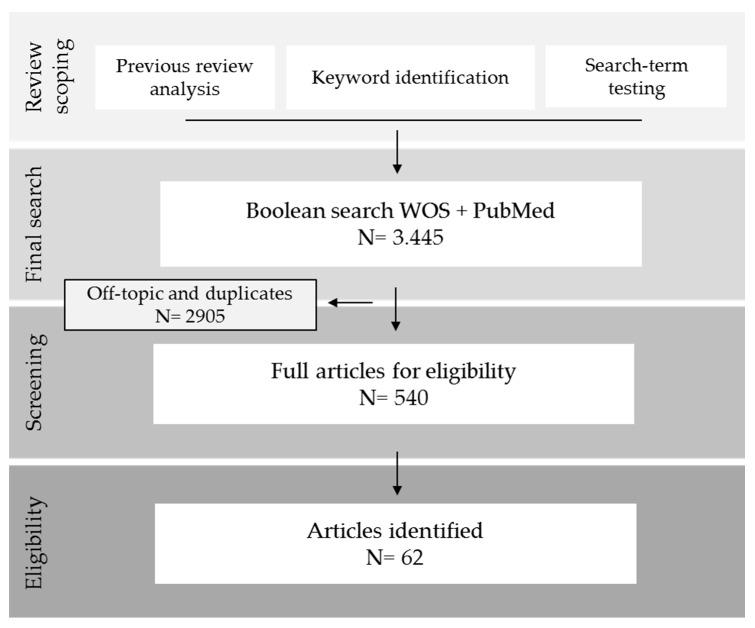
Flow diagram of article selection for the systematic review.

The combined searches identified 3445 articles (Figure 1). Titles, abstracts and keywords were screened, and duplicates and off-topic articles were excluded. Only articles published between 1900 and 2019 and correlating any forest (including urban or virtually imaged) with any variable of human health or well-being were eligible. 

### 2.4. Eligibility

Articles were examined for title, abstract, keywords and methods, if needed. Inclusion considered the following criteria:

- experiments with a real-life exposure to forests;
- experimental studies with humans;
- articles containing quantitative objective measurements of physiological health variables.

Laboratory experiments and studies with only virtual images or videos; studies with qualitative, self-rated or self-perceived health issues; reviews and articles using broad-scale spatial data were all excluded. In this study forests are considered as areas covered by trees which are not predominantly under agricultural or urban land use. Therefore, we did not include studies conducted in plantations or urban forests. A total of 62 articles were ultimately included in the analysis. 

### 2.5. Data Collection

Three authors gathered the information from each article using a codebook, with consensual definitions and consultation to resolve differences. Inter-code consistency was tested by each author by reviewing three random articles. 

Details of variables were collected from each study based on article basics, exposure characteristics, forest descriptions and health outcomes. Items from each group are presented below.

- Article basics: *authors*, *year* of publication, *title*, *country* where the study was conducted, *number of participants* and *study design*. This last item, concerning the methodology used in the study, was further classified into three categories: before-after (B-A) studies, control trials (CTs) (randomized or not) and comparative studies (when different forests or types of groups were compared). This distinction was made to better register the records of health variables for a more comprehensive analysis. Effects before and after forest exposure, between urban and forest environments or even between forest types could thus be compared.
- Exposure characteristics: we suggested the following *exposure type* classification considering three levels: (a) passive exposure (when participants just stayed in the forest, sitting or viewing the landscape, or passively walked around the forest); (b) active exposure (when participants did any kind of moderate to vigorous-intense physical activity such as running, cycling or any other sport) and (c) pro-active exposure (when the activities and actions conducted could induce mental or physical well-being effects by itself, without necessarily being in the forest, e.g., yoga, meditation). *Exposure time* was also recorded and ranked in minutes (≤60 min), hours (≤24 h), days (≤365 days) and years (>1 year).
- Forest descriptions: forest information from the articles were gathered and sorted into eight categories: *forest type* (taken from the species composition or dominance if not explicitly mentioned in the text), *abiotic variables measured* (e.g., temperature, humidity, light intensity, wind speed), *general forest description* (referring to any broad information about the forest, such as the surface of the forested area, the conservation status or any geographical information like the altitude), *forest species described* (tree species composition), *forest age*, *management strategy* (any information about the management regime develop in the studied forest), *forest variables described* (e.g., tree density, biodiversity, BVOCs, vegetation structure, diameter at breast height, basal area) and *forest variables measured* (if numerical values of the measurements were recorded). All data were gathered into these representative categories of the detail degree of description for a forest ecosystem. We assumed forest type as the broadest description of a forest, and the measurement of a particular forest variable as the most accurate and precise approach for describing a stand.
- Health outcomes: measured health variables were registered for each article. The effects of forests in B-A studies were assessed and classified at four levels: *increases* (if the value of a variable increased significantly after a visit to the forest), *decreases* (if the value of a variable decreased significantly after a visit to the forest), *non-significant* (NS) results after the trial and *mixed effects* (when changes were significant but increased for some participants while decreased for others). Similarly, the effect of a forest was assessed for CTs and classified as: *Higher* (if the value of a variable was significantly higher in the forest than the urban group), *Lower* (if the value of a variable was significantly lower in the forest than the urban group), *Non-significant* (NS) after the trial and *Mixed effects* (when changes were significant but were lower for some participants and higher for others). Significant levels were set at *p* < 0.05. Health variables were not registered if an article did not explicitly present statistical analysis of values before and after exposure or a comparison between the tested environments.

### 2.6. Data Analysis

Descriptive and exploratory analyses were used to identify patterns among the data collected. We analyzed the frequency in which different forest variables were described in the existing literature sorting them out in categories according to the degree of detail description and produced summary statistics. We developed pivot tables to examine the relationships between forest and human health variables. These relationships were obtained only for the most commonly studied health variables registered in this review: blood pressure (diastolic and systolic), pulse rate and cortisol levels (blood and saliva). These variables were also surveyed in two recent reviews, which found strong correlations with exposure to forest ecosystems [5,8]. 

## 3. Results

### 3.1. General Overview

An overview of the selected articles’ basics is presented in Table S1. Ninety percent of the articles were conducted in Asian countries (53.23% of the total in Japan, 19.35% in South Korea, 12.90% in China and 4.84% in Taiwan). Only six of the 62 studies were conducted in Europe (three in Sweden and one each in Poland, Denmark and Spain). The number of participants involved in the studies varied from seven [19] to 625 [20]. Exposure time also varied greatly among the studies, from 10 min to repeated exposures for six years. Most of the articles (59.68% of the total) considered short exposure times (minutes or hours), whereas 40.32% investigated the health impact of one day or longer exposures. For exposure type, 79.03% of the articles analyzed the effects of forests during a passive exposure to a forest environment (motionless, viewing or passively walking only), 20.97% studied the effect of a pro-active exposure (e.g., yoga, mindfulness) and none of the articles assessed the effects during an active exposure (doing any intense physical activity). Of the total, 64.52% were CTs, 24.19% were B-A tests and the remainder were comparative studies. 

### 3.2. Forest Variables

We found no consistent and uniform consensus between the descriptions of the forest ecosystems and human health (Table 1). The forest where the analyzed studies were conducted was not described in 12 out of the 62 selected articles. Considering forest type, 33.87% of the studies did not provide this information, even though our requirements for this item description were broad. The forest types studied in the articles were: coniferous, broad-leaved (evergreen and deciduous), bamboo and mixed forests. Forest age, which varied from 10 to 120 years, was reported in 10 articles. Seventeen articles provided information for described or measured forest variables. From two articles providing the management regime of the studied forest, only one described the technique applied in the stand. The specific forest variables identified were: tree density (five articles), diameter at breast height (two articles), biodiversity level (one article), species dominance (one article), tree height (one article), BVOCs concentrations (five articles), air quality (four articles) and pollutant concentrations (one article). The main BVOCs identified by the five studies that reported concentrations were alpha- and beta-pinene, tricyclene, camphene, limonene, camphor, alpha-phellandrene, carene and isoprene [21,22,23,24,25].

### 3.3. Health Variables

Among the 62 studies, 103 health variables were recorded (Table S2), including measurements of the same variable in different samples (e.g., blood, urine, saliva). The measured variables mainly belonged to the endocrine/reproductive, cardiovascular, metabolic, nervous, respiratory and immune systems. The most commonly studied health variables were blood pressure (23 B-A and 20 CT records on diastolic blood pressure (DBP) and 24 B-A and 18 CT records on systolic blood pressure (SBP) from all selected articles), pulse rate (PR) (12 B-A and 9 CT records among all selected articles) and cortisol levels (11 B-A and CT records in studies measuring blood and saliva levels from all selected articles). 

Nearly 55% of the studies provided information about the potential effect of forest type on blood pressure (Table 2 and Table 3). Mixed forests were studied the most for both DBP and SBP in B-A studies, where only one study reported an increase in SBP after forest exposure. The DBP records showed no consistent patterns, specifically referring to the effects of mixed forests (six non-significant records vs three records of decreases). For SBP, more consistency can be observed with six decrease records vs two non-significant. Only single decrease records were identified in coniferous and bamboo forests for both blood pressures. 

Contrasting these records with CTs measurements in the cities (Table 3) indicated that the blood-pressure levels were not higher in forests than the cities in any of these studies, although one study reported mixed effects among participants in a broad-leaved forest. Three records complemented the contribution of coniferous forests to blood pressure, with lower levels after forest exposure compared to city exposure for DBP. In contrast, the previous B-A decrease records for mixed forests were not supported by the CTs records, where only one DBP record was lower for forest than city, whereas the rest indicated non-significant differences between the two environments. 

The effects on PR are presented in Table 4. The B-A records did not follow a specific pattern. CTs records of PR, however, were lower in three studies comparing mixed forests to cities. 

Few of the B-A studies reported cortisol levels. Cortisol measurements were reported for only two forest types: broad-leaved and mixed forests (Table 5). The B-A studies did not identify a decrease in cortisol levels for broad-leaved forests, but levels were lower for broad-leaved forests than cities. Likewise, cortisol levels were consistently lower in the B-A and CT records for mixed forest, even when compared to cities. 

## 4. Discussion

This systematic review was conducted to analyze the details of forest descriptions among studies of these ecosystems’ potential effects on human health and to identify patterns between forest and health variables, when possible. The number of participants, exposure time and type were highly variable among the 62 selected articles. The impacts on health were generally positive, while a considerable number of studies showed non-significant results. Most studies addressed physiological health variables during short periods of time in small samples of healthy populations mainly focusing on parameters with low specificity for clinical decisions. 

This review found that: (a) 19.35% of the articles lacked any forest description; (b) the descriptions in the articles that did provide this information were highly heterogeneous and (c) no pattern was identified in the data between health variables from the three most studied variables (blood pressure, PR and cortisol level) and the basic level of forest description considered: forest type. From the total, 66.13% of the articles described the forest type or procured information to estimate it. Other reviews analyzing the link between nature, forests and human health have also reported this scarcity of confounding factors description of forest environments [17,78], perhaps due to the belief that these data are not yet relevant. Descriptions of forest ecosystems for determining effects on human health may nevertheless become crucial with the increasing interest from many spheres of the society. This topic has both attracted the attention of the scientific community in recent decades [1,2] and has become an emerging priority for policy makers, managers and planners. Examples can be found at international level, where the use of forests to foster human health has been included in the agendas of forest policy (e.g., IUFRO Task Force on Forest and human Health, 2007), in Asian countries such as Japan and South Korea [1,14] and in some European countries [7]. Some studies predict that forest priorities can change from production and conservation to recreation and health promotion [14,15], so detailed information about the characteristics of these habitats should be provided for its management and its potential use for preventive medicine. 

Some studies have begun to address the relevance of forest characteristics to human health. Saito et al. (2019) assessed the differences in various health variables between exposure to an unmanaged forest and a managed forest [26]. Blood pressure and saliva cortisol levels decreased significantly for both forests after a stress stimulus but records were significantly lower in the managed than the unmanaged forest [26]. Another study reported that health responses differed between an unmanaged and a managed forest, with significantly more favorable acute insulin reactions and levels of oxidative stress in the unmanaged forest, underlying more profound beneficial effects in the unmanaged than the managed forest [25]. These two studies, however, did not use a control group in an urban setting. Sonntag-Öström et al. (2014) reported that heart rate was significantly lower for three forest environments (a forest by a lake, an open forest with exposed bedrock and a closed spruce forest) when compared to a city and in particular, it was significantly lower for the forest by the lake than the other forest environments [39]. DBP in the same study did not differ significantly between the city and the forest with exposed bedrock but was significantly lower for the forest by the lake and the spruce forest than the city [39]. An et al. (2004) analyzed through digital images the different effects of stand density for two forest types, showing higher frontal brain activity related to greater stand density and brain relaxation when viewing lower stand density in coniferous forests while for the broad-leaved forest images, 50% stand density was related to stability of brain activity and PR, whereas 100% density was associated with more active electroencephalogram [79]. Blood pressure, heart rate, body temperature and oxygen saturation did not differ significantly before and after forest exposure in a comparative study contrasting the health effects of a mature and a young forest [37]. The results of these studies together with our findings indicate the need for further research and the need to integrate descriptions and measurements of forest characteristics in studies of human health. 

In regard to forest variables, some studies have identified BVOCs as a key element in these ecosystems [12,25,80]. Terpenes, sometimes called phytoncides, are the largest class of naturally occurring organic compounds and the major components of forest atmospheres [81]. These compounds are produced by plants as a defensive mechanism against environmental stress and herbivory [81,82,83]. Terpenes that relevantly affect cellular and animal systems have shown anti-inflammatory, anti-tumorigenic or neuroprotective activities [13]. Only five of the studies in our review measured terpene levels in forest air, identifying mainly alpha- and beta-pinene, tricyclene, camphene, limonene, camphor, alpha-phellandrene, carene and isoprene [21,22,23,24,25]. Lee et al. (2018) significantly associated alpha-phellandrene with an acute insulin reaction, consistent with another in vivo study where alpha-phellandrene increased immune responses [84]. Although the included studies of Dr. Li did not directly associate the health outcomes observed with terpenes, a previous in vitro study did, reporting a significant increase in human natural killer (NK) cells activity and in the expression of intracellular cytolytic molecules, perforin, granzyme A (GrA) and granulysin (GRN) by phytoncides [11]. Komori et al. (1995) similarly reported the effects of citrus fragrance in forests on the immune and endocrine systems, analyzing NK cells activity and urinary cortisol and dopamine levels [85]. Li et al. (2009) reported significantly higher NK activity and percentages of NK, perforin, granulysin and granzyme A/B-expressing cells, as well as significantly lower percentage of T cells and concentrations of adrenaline and noradrenaline in urine when phytoncides were vaporized in hotel rooms at night [12]. These findings together indicate that increased NK activity in subjects visiting a forest may be partially due to forest terpenes [21,22]. Another study found that inhaling oils containing terpenes significantly decreased SBP, DBP and cortisol levels [86]. Surprisingly, as far as we know, only one study has analyzed the absorption of terpenes in blood after forest exposure [87]. The authors of this study identified the monoterpenes species present in coniferous-forest atmosphere in serum samples of the subjects who were walking in the forest [87]. They also identified an increase in the amount of alpha-pinene in the serum after the subjects walked in the forest as well as differences in monoterpene composition and abundance between coniferous and broad-leaved forests air [87]. Lee et al. (2018) reported that the mean atmospheric concentration of phytoncides was higher in a natural than a tended forest (25.58 vs 18.44 ng/m^3^, respectively) [25]. Studies are increasingly providing evidence of the role of these compounds in human health, but more research is needed to describe and predict the composition and abundance of terpenes under forest canopies, to analyze the absorption and metabolism of these chemicals by humans and for identifying the mechanisms leading to health effects. 

This review could not find any pattern in the data between the three most studied health variables (blood pressure, pulse rate and cortisol level) that could account for the basic level of forest description considered: forest type. These findings are similar to those of other reviews that considered the effects of exposure to nature on mental health [78] or the benefits of forests to health and well-being [17]. Although not including any forest variable in the analysis, blood pressure and cortisol levels have been surveyed by two recent systematic reviews which conducted meta-analyses and significantly linked forest exposure with the decreases of these health variables [5,8]. Thus, the scarce descriptions of forest variables identified in our review that could affect objectively measurable physiological health variables may therefore apply generally. We identified a weak relationship in SBP records among B-A trials showing a decrease in mixed forests. This was not supported by the analyzed control trials and might be due to the broaden categorization of these forest type. Encouraging future research to develop a more integrative approach is thus essential, both analyzing the effects of forests and characterizing ecosystems to describe the pathways that may induce health effects to provide data and tools for forest managers, policy makers and planners in coordination with healthcare professionals. 

## 5. Limitations

The limitations of language may have been important when developing the systematic search because many of the publications were in Asian languages. The high variability of participant number, exposure time, forest type and study design may have induced analytical biases among the data collected. The assumption of forest type when tree composition or dominance were provided may also have added biases to the analysis due to the scarcity of basic forest descriptions. We were also conscious of the challenge to identify patterns and relationships by analyzing studies of real-life exposure to natural forested areas where subject reactions and health effects may be influenced by different sensory stimuli, seasonal variabilities, meteorological conditions, exposures and performances of the activities conducted or other inputs not previously considered. Only three health variables among the studies could be screened. Nevertheless, other health variables not considered in this review may also be relevant to human health in future studies. These in situ studies nevertheless provided relevant data for understanding the connection between human health and forest ecosystems, even though the effects of forests on humans may presumably be derived from different inputs, components and characteristics of forest environments.

## 6. Conclusions

This systematic review underlines the lack of forest variables descriptions among studies of forests and human health and highlights that it is still premature to make any sort of conclusions with respect to data patterns due to the high heterogeneity within the studies performed so far. Furthermore, no consistent relationships between forest type and health variables (blood pressure, pulse rate and cortisol levels) could be identified from the existing literature.

## Figures and Tables

**Table 1:** Details of forest descriptions. Symbol “*” indicates that the article provides the forest description information. Manag. Strategy, management strategy; Forest var. described, forest variables described; and Forest var. measured, forest variables measured.

Reference	(−) Detail of Description (+)							
	Forest Type	Abiotic Variables	Forest Description	Forest Species	Forest Age	Manag. Strategy	Forest Var. Described	Forest Var. Measured
[26]	*	*	*	*	*	*	*	*
[25]	*	*	*	*	*		*	*
[27]	*	*	*	*	*		*	*
[28]	*	*	*	*	*		*	*
[29]	*	*	*	*			*	*
[30]	*	*	*	*			*	*
[31]	*	*	*	*			*	*
[32]	*	*	*	*			*	*
[33]	*	*	*	*			*	*
[34]	*	*		*			*	*
[22]	*	*		*			*	*
[35]	*	*	*				*	*
[24]	*			*			*	*
[36]			*				*	*
[23]	*	*					*	*
[21]		*					*	*
[37]	*	*	*	*	*		*	
[38]	*	*		*		*		
[39]	*	*	*	*	*			
[18]	*	*		*	*			
[40]	*	*		*	*			
[41]	*		*	*	*			
[42]	*				*			
[43]	*	*	*	*				
[44]	*	*	*	*				
[45]	*	*	*	*				
[46]	*	*	*	*				
[47]	*	*	*	*				
[48]	*	*	*	*				
[49]	*	*	*	*				
[50]	*		*	*				
[51]	*	*		*				
[52]	*	*						
[53]	*	*		*				
[54]	*	*		*				
[55]	*		*	*				
[56]	*			*				
[57]	*		*	*				
[58]	*			*				
[59]	*			*				
[60]		*	*					
[61]		*	*					
[62]		*	*					
[63]			*					
[64]		*						
[4]		*						
[65]		*						
[66]	*							
[2]	*							
[67]	*							
[68]								
[69]								
[19]								
[70]								
[71]								
[72]								
[73]								
[74]								
[75]								
[20]								
[76]								
[77]								

**Table 2:** Effects of forest type on blood pressure in the before-after studies. (A) diastolic blood pressure (DBP) and (B) systolic blood pressure (SBP). NS, not significant.

**A**	**Forest Type**	***Effect on Diastolic Blood Pressure (DBP)***				
		**Decrease**	**NS**	**Increase**	**Mixed Effects**	**Total**
	Broad-leaved	-	1	-	1	2
	Coniferous	1	-	-	-	1
	Bamboo	1	-	-	-	1
	Mixed	3	6	-	-	9
	Total	5	7	-	1	13
B		*Effect on Systolic Blood Pressure (SBP)*				
		Decrease	NS	Increase	Mixed effects	Total
	Broad-leaved	-	2	-	-	2
	Coniferous	1	-	-	-	1
	Bamboo	1	-	-	-	1
	Mixed	6	2	1	-	9
	Total	8	4	1	-	13

**Table 3:** Effects of forest type on blood pressure in the control trials. (A) Diastolic blood pressure (DBP) and (B) systolic blood pressure (SBP). NS, not significant.

**A**	**Forest Type**	***Effect on Diastolic Blood Pressure (DBP)***				
		**Lower**	**NS**	**Higher**	**Mixed Effects**	**Total**
	Broad-leaved	1	1	-	1	3
	Coniferous	3	-	-	-	3
	Bamboo	1	-	-	-	1
	Mixed	1	5	-	-	6
	Total	6	6	-	1	13
B		*Effect on Systolic Blood Pressure (SBP)*				
		Lower	NS	Higher	Mixed effects	Total
	Broad-leaved	1	1	-	1	3
	Coniferous	1	1	-	-	2
	Bamboo	-	1	-	-	1
	Mixed	-	6	-	-	6
	Total	2	9	-	1	12

**Table 4:** Effects of forest type on pulse rate (PR) in the (A) before-after studies and the (B) control trials. NS, not significant.

**A**	**Forest Type**	***Effect on Pulse Rate***				
		**Decrease**	**NS**	**Increase**	**Mixed Effects**	**Total**
	Broad-leaved	-	-	-	1	1
	Mixed	2	3	1	-	6
	Total	2	3	1	1	7
B		Lower	NS	Higher	Mixed effects	Total
	Broad-leaved	1	1	-	-	2
	Coniferous	1	-	-	-	1
	Mixed	3	-	-	-	3
	Total	5	1	-	-	6

**Table 5:** Effects of forest type on cortisol levels in the (A) before-after studies and the (B) control trials. NS, not significant.

**A**	**Forest Type**	***Effect on Cortisol Levels***				
		**Decrease**	**NS**	**Increase**	**Mixed Effects**	**Total**
	Broad-leaved	-	2	-	-	2
	Mixed	2	-	-	-	2
	Total	2	2	-	-	4
B		Lower	NS	Higher	Mixed effects	Total
	Broad-leaved	4	1	-	-	5
	Mixed	3	-	-	-	3
	Total	7	1	-	-	8

## Supplementary Materials

The following are available online at https://www.mdpi.com/1660-4601/17/3/1027/s1, Table S1: Overview of the analyzed articles basic information, Table S2: Health variables registered in the articles analyses classified by systems or functions.

## References

[1] Tsunetsugu Y., Park B.J., Miyazaki Y. (2010). Trends in research related to “shinrin-yoku” (taking in the forest atmosphere or forest bathing) in Japan. Environ. Health Prev. Med..

[2] Lee J., Park B.J., Tsunetsugu Y., Ohira T., Kagawa T., Miyazaki Y. (2011). Effect of forest bathing on physiological and psychological responses in young Japanese male subjects. Public Health.

[3] Song C., Ikei H., Miyazaki Y. (2016). Physiological effects of nature therapy: A review of the research in Japan. Int. J. Environ. Res. Public Health.

[4] Park B.J., Tsunetsugu Y., Kasetani T., Kagawa T., Miyazaki Y. (2010). The physiological effects of Shinrin-yoku (taking in the forest atmosphere or forest bathing): Evidence from field experiments in 24 forests across Japan. Environ. Health Prev. Med..

[5] Ideno Y., Hayashi K., Abe Y., Ueda K., Iso H., Noda M., Lee J.-S., Suzuki S. (2017). Blood pressure-lowering effect of Shinrin-yoku (Forest bathing): A systematic review and meta-analysis. BMC Complement. Altern. Med..

[6] Meyer K., Bürger-Arndt R. (2014). How forests foster human health–Present state of research-based knowledge (in the field of Forests and Human Health). Int. For. Rev..

[7] Meyer-Schulz K., Bürger-Arndt R. (2019). Les effets de la forêt sur la santé physique et mentale. Une revue de la littérature scientifique. Rev. For. Française.

[8] Antonelli M., Barbieri G., Donelli D. (2019). Effects of forest bathing (shinrin-yoku) on levels of cortisol as a stress biomarker: A systematic review and meta-analysis. Int. J. Biometeorol..

[9] Hansen M.M., Jones R., Tocchini K. (2017). Shinrin-yoku (Forest bathing) and nature therapy: A state-of-the-art review. Int. J. Environ. Res. Public Health.

[10] Morita E., Fukuda S., Nagano J., Hamajima N., Yamamoto H., Iwai Y., Nakashima T., Ohira H., Shirakawa T. (2007). Psychological effects of forest environments on healthy adults: Shinrin-yoku (forest-air bathing, walking) as a possible method of stress reduction. Public Health.

[11] Li Q., Nakadai A., Matsushima H., Miyazaki Y., Krensky A., Kawada T., Morimoto K. (2006). Phytoncides (wood essential oils) induce human natural killer cell activity. Immunopharmacol. Immunotoxicol..

[12] Li Q., Kobayashi M., Wakayama Y., Inagaki H., Katsumata M., Hirata Y., Hirata K., Shimizu T., Kawada T., Park B.J. (2009). Effect of phytoncide from trees on human natural killer cell function. Int. J. Immunopathol. Pharmacol..

[13] Cho K.S., Lim Y.R., Lee K., Lee J., Lee J.H., Lee I.S. (2017). Terpenes from forests and human health. Toxicol. Res..

[14] Shin W.S., Kim J., Lim S.S., Yoo R., Jeong M., Park S. (2017). Paradigm shift on forest utilization: Forest service for health promotion in the Republic of Korea. NET J. Agric. Sci..

[15] Kim G., Park B.J., Joung D., Yeom D.G., Koga S. (2015). Healing environments of major tree species in Kyushu University forests: A case study. J. Fac. Agric. Kyushu Univ..

[16] Collaboration for Environmental Evidence Guidelines for Systematic Review and Evidence Synthesis in Environmental Management. www.environmentalevidence.org/Documents/Guidelines/Guidelines4.2.pdf.

[17] Oh B., Lee K.J., Zaslawski C., Yeung A., Rosenthal D., Larkey L., Back M. (2017). Health and well-being benefits of spending time in forests: Systematic review. Environ. Health Prev. Med..

[18] Tsunetsugu Y., Park B.-J., Ishii H., Hirano H., Kagawa T., Miyazaki Y. (2007). Physiological Effects of Shinrin-yoku (Taking in the Atmosphere of the Forest) in an Old-Growth Broadleaf Forest in Yamagata Prefecture, Japan. J. Physiol. Anthropol..

[19] Joung D., Kim G., Choi Y., Lim H., Park S., Woo J.M., Park B.J. (2015). The prefrontal cortex activity and psychological effects of viewing forest landscapes in Autumn season. Int. J. Environ. Res. Public Health.

[20] Kobayashi H., Song C., Ikei H., Kagawa T., Miyazaki Y. (2015). Analysis of Individual Variations in Autonomic Responses to Urban and Forest Environments. Evid.-Based Complement. Altern. Med..

[21] Li Q., Morimoto K., Nakadai A., Inagaki H., Katsumata M., Shimizu T., Hirata Y., Hirata K., Suzuki H., Miyazaki T. (2007). Forest Bathing Enhances Human Natural Killer Activity and Expression of Anti-Cancer Proteins. Int. J. Immunopathol. Pharmacol..

[22] Li Q., Morimoto K., Kobayashi M., Inagaki H., Katsumata M., Hirata Y., Hirata K., Shimizu T., Li Y.J., Wakayama T. (2008). A forest bathing trip increases human natural killer activity and expression of anti-cancer proteins in female subjects. J. Biol. Regul. Homeost. Agents.

[23] Li Q., Morimoto K.I., Kobayashi M., Inagaki H., Katsumata M., Hirata Y., Hirata K., Suzuki H., Li Y., Wakayama Y. (2008). Visiting a Forest, but not a City, Increases Human Natural Killer Activity and Expression of Anti-Cancer Proteins cells, and intracellular anti-cancer proteins in lymphocytes. In the present study, we investigated how with a trip to places in a city. Int. J. Immunopathol. Pharmacol..

[24] Im S.G., Choi H., Jeon Y.H., Song M.K., Kim W., Woo J.M. (2016). Comparison of effect of two-hour exposure to forest and urban environments on cytokine, anti-oxidant, and stress levels in young adults. Int. J. Environ. Res. Public Health.

[25] Lee K.J., Hur J., Yang K.S., Lee M.K., Lee S.J. (2018). Acute Biophysical Responses and Psychological Effects of Different Types of Forests in Patients With Metabolic Syndrome. Environ. Behav..

[26] Saito H., Horiuchi M., Takayama N., Fujiwara A. (2019). Effects of managed forest versus unmanaged forest on physiological restoration from a stress stimulus, and the relationship with individual traits. J. For. Res..

[27] Bielinis E., Bielinis L., Krupińska-Szeluga S., Łukowski A., Takayama N. (2019). The effects of a short forest recreation program on physiological and psychological relaxation in young polish adults. Forests.

[28] Yu C.P., Lin C.M., Tsai M.J., Tsai Y.C., Chen C.Y. (2017). Effects of short forest bathing program on autonomic nervous system activity and mood states in middle-aged and elderly individuals. Int. J. Environ. Res. Public Health.

[29] Mao G.X., Cao B.Y., Yang Y., Chen Z.M., Dong J.H., Chen S.S., Wu Q., Lyu X.L., Jia B.B., Yan J. (2018). Additive benefits of twice forest bathing trips in elderly patients with chronic heart failure. Biomed. Environ. Sci..

[30] Mao G., Cao Y., Wang B., Wang S., Chen Z., Wang J., Xing W., Ren X., Lv X., Dong J. (2017). The salutary influence of forest bathing on elderly patients with chronic heart failure. Int. J. Environ. Res. Public Health.

[31] Mao G.-X., Cao Y.-B., Lan X.-G., He Z.-H., Chen Z.-M., Wang Y.-Z., Hu X.-L., Lv Y.-D., Wang G.-F., Yan J. (2012). Therapeutic effect of forest bathing on human hypertension in the elderly. J. Cardiol..

[32] Wu Q., Cao Y., Mao G., Wang S., Fang Y., Tong Q., Huang Q., Wang B., Yan J., Wang G. (2017). Effects of forest bathing on plasma endothelin-1 in elderly patients with chronic heart failure: Implications for adjunctive therapy. Geriatr. Gerontol. Int..

[33] Shin J.-W., Choi J.-H. (2019). The effects of single session forest walking on physiological and psychological state of myocardial infarction patients. J. People Plants Environ..

[34] Horiuchi M., Endo J., Takayama N., Murase K., Nishiyama N., Saito H., Fujiwara A. (2014). Impact of viewing vs. not viewing a real forest on physiological and psychological responses in the same setting. Int. J. Environ. Res. Public Health.

[35] Tsao T.-M., Tsai M.-J., Hwang J.-S., Cheng W.-F., Wu C.-F., Chou C.-C., Su T.-C. (2018). Health effects of a forest environment on natural killer cells in humans: An observational pilot study. Oncotarget.

[36] Yamaguchi M., Deguchi M., Miyazaki Y. (2006). The effects of exercise in forest and urban environments on sympathetic nervous activity of normal young adults. J. Int. Med. Res..

[37] López-Pousa S., Bassets Pagès G., Monserrat-Vila S., de Gracia Blanco M., Hidalgo Colomé J., Garre-Olmo J. (2015). Sense of well-being in patients with fibromyalgia: Aerobic exercise program in a mature forest—A pilot study. Evid. Based Complement. Altern. Med..

[38] Dolling A., Nilsson H., Lundell Y. (2017). Stress recovery in forest or handicraft environments—An intervention study. Urban For. Urban Green..

[39] Sonntag-Öström E., Nordin M., Lundell Y., Dolling A., Wiklund U., Karlsson M., Carlberg B., Slunga Järvholm L. (2014). Restorative effects of visits to urban and forest environments in patients with exhaustion disorder. Urban For. Urban Green..

[40] Park B.J., Tsunetsugu Y., Kasetani T., Hirano H., Kagawa T., Sato M., Miyazaki Y. (2007). Physiological effects of Shinrin-yoku (taking in the atmosphere of the forest) using salivary cortisol and cerebral activity as indicators. J. Physiol. Anthropol..

[41] Sung J., Woo J.M., Kim W., Lim S.K., Chung E.J. (2012). The effect of cognitive behavior therapy-based “forest therapy” program on blood pressure, salivary cortisol level, and quality of life in elderly hypertensive patients. Clin. Exp. Hypertens..

[42] Park B.J., Tsunetsugu Y., Kasetani T., Morikawa T., Kagawa T., Miyazaki Y. (2009). Physiological effects of forest recreation in a young conifer forest in Hinokage Town, Japan. Silva Fenn..

[43] Sonntag-Öström E., Nordin M., Dolling A., Lundell Y., Nilsson L., Slunga Järvholm L. (2015). Can rehabilitation in boreal forests help recovery from exhaustion disorder? The randomised clinical trial ForRest. Scand. J. For. Res..

[44] Song C., Ikei H., Miyazaki Y. (2017). Sustained effects of a forest therapy program on the blood pressure of office workers. Urban For. Urban Green..

[45] Jia B.B., Yang Z.X., Mao G.X., Lyu Y.D., Wen X.L., Xu W.H., Lyu X.L., Cao Y.B., Wang G.F. (2016). Health Effect of Forest Bathing Trip on Elderly Patients with Chronic Obstructive Pulmonary Disease. Biomed. Environ. Sci..

[46] Lee J.Y., Lee D.C. (2014). Cardiac and pulmonary benefits of forest walking versus city walking in elderly women: A randomised, controlled, open-label trial. Eur. J. Integr. Med..

[47] Wang D.H., Yamada A., Miyanaga M. (2018). Changes in urinary hydrogen peroxide and 8-hydroxy-2′-deoxyguanosine levels after a forest walk: A pilot study. Int. J. Environ. Res. Public Health.

[48] Chen H.T., Yu C.P., Lee H.Y. (2018). The effects of forest bathing on stress recovery: Evidence from middle-aged females of Taiwan. Forests.

[49] Song C., Ikei H., Kagawa T., Miyazaki Y. (2019). Effects of walking in a forest on young women. Int. J. Environ. Res. Public Health.

[50] Lee J., Tsunetsugu Y., Takayama N., Park B.-J., Li Q., Song C., Komatsu M., Ikei H., Tyrväinen L., Kagawa T. (2014). Influence of forest therapy on cardiovascular relaxation in young adults. Evid.-Based Complement. Altern. Med..

[51] Han J.-W., Choi H., Jeon Y.-H., Yoon C.-H., Woo J.-M., Kim W. (2016). The effects of forest therapy on coping with chronic widespread pain: Physiological and psychological differences between participants in a forest therapy program and a control group. Int. J. Environ. Res. Public Health.

[52] Hassan A., Tao J., Li G., Jiang M., Aii L., Zhihui J., Zongfang L., Qibing C. (2018). Effects of walking in bamboo forest and city environments on brainwave activity in young adults. Evid.-Based Complement. Altern. Med..

[53] Lee J., Park B.J., Tsunetsugu Y., Kagawa T., Miyazaki Y. (2009). Restorative effects of viewing real forest landscapes, based on a comparison with urban landscapes. Scand. J. For. Res..

[54] Song C., Ikei H., Kobayashi M., Miura T., Li Q., Kagawa T., Kumeda S., Imai M., Miyazaki Y. (2017). Effects of viewing forest landscape on middle-aged hypertensive men. Urban For. Urban Green..

[55] Yu Y.M., Lee Y.J., Kim J.Y., Yoon S.B., Shin C.S. (2016). Effects of forest therapy camp on quality of life and stress in postmenopausal women. Forest Sci. Technol..

[56] Song C., Ikei H., Kobayashi M., Miura T., Taue M., Kagawa T., Li Q., Kumeda S., Imai M., Miyazaki Y. (2015). Effect of forest walking on autonomic nervous system activity in middle-aged hypertensive individuals: A pilot study. Int. J. Environ. Res. Public Health.

[57] Seo S.C., Park S.J., Park C.-W., Yoon W.-S., Choung J.T., Yoo Y. (2015). Clinical and immunological effects of a forest trip in children with asthma and atopic dermatitis. Iran. J. Allergy Asthma Immunol..

[58] Mao G.X., Lan X.G., Cao Y.B., Chen Z.M., He Z.H., LV Y.D., Wang Y.Z., Hu X.L., Wang G.F., Yan J. (2012). Effects of short-term forest bathing on human health in a broad-leaved evergreen forest in Zhejiang Province, China. Biomed. Environ. Sci..

[59] Chun M.H., Chang M.C., Lee S.J. (2017). The effects of forest therapy on depression and anxiety in patients with chronic stroke. Int. J. Neurosci..

[60] Ochiai H., Ikei H., Song C., Kobayashi M., Miura T., Kagawa T., Li Q., Kumeda S., Imai M., Miyazaki Y. (2015). Physiological and psychological effects of a forest therapy program on middle-aged females. Int. J. Environ. Res. Public Health.

[61] Ochiai H., Ikei H., Song C., Kobayashi M., Takamatsu A., Miura T., Kagawa T., Li Q., Kumeda S., Imai M. (2015). Physiological and psychological effects of forest therapy on middle-aged males with high-normal blood pressure. Int. J. Environ. Res. Public Health.

[62] Li Q., Otsuka T., Kobayashi M., Wakayama Y., Inagaki H., Katsumata M., Hirata Y., Li Y., Hirata K., Shimizu T. (2011). Acute effects of walking in forest environments on cardiovascular and metabolic parameters. Eur. J. Appl. Physiol..

[63] Stigsdotter U.K., Corazon S.S., Sidenius U., Kristiansen J., Grahn P. (2017). It is not all bad for the grey city—A crossover study on physiological and psychological restoration in a forest and an urban environment. Heal. Place.

[64] Ohtsuka Y., Yabunaka N., Takayama S. (1998). Shinrin-yoku (forest-air bathing and walking) effectively decreases blood glucose levels in diabetic patients. Int. J. Biometeorol..

[65] Li Q., Otsuka T., Kobayashi M., Wakayama Y., Inagaki H., Katsumata M., Hirata Y., Li Y., Hirata K., Shimizu T. (2016). Effects of forest environments on cardiovascular and metabolic parameters. J. Evid.-Based Complement. Altern. Med..

[66] Bang K.S., Lee I., Kim S., Lim C.S., Joh H.K., Park B.J., Song M.K. (2017). The effects of a campus Forest-Walking program on undergraduate and graduate students’ physical and psychological health. Int. J. Environ. Res. Public Health.

[67] Park B.J., Tsunetsugu Y., Ishii H., Furuhashi S., Hirano H., Kagawa T., Miyazaki Y. (2008). Physiological effects of Shinrin-yoku (taking in the atmosphere of the forest) in a mixed forest in Shinano Town, Japan. Scand. J. For. Res..

[68] Ohe Y., Ikei H., Song C., Miyazaki Y. (2017). Evaluating the relaxation effects of emerging forest-therapy tourism: A multidisciplinary approach. Tour. Manag..

[69] Horiuchi M., Endo J., Akatsuka S., Uno T., Hasegawa T. (2013). Influence of forest walking on blood pressure, profile of mood states and stress markers from the viewpoint of aging. J. Aging Gerontol..

[70] Morita E., Imai M., Okawa M., Miyaura T., Miyazaki S. (2011). A before and after comparison of the effects of forest walking on the sleep of a community-based sample of people with sleep complaints. Biopsychosoc. Med..

[71] Toda M., Takeshita T. (2015). The influence of personal patterns of behavior on the physiological effects of woodland walking. Adv. Mind Body Med..

[72] Toda M., Den R., Hasegawa-Ohira M., Morimoto K. (2013). Effects of woodland walking on salivary stress markers cortisol and chromogranin A. Complement. Ther. Med..

[73] Song C., Ikei H., Lee J., Park B.-J., Kagawa T., Miyazaki Y. (2013). Individual differences in the physiological effects of forest therapy based on Type A and Type B behavior patterns. J. Physiol. Anthropol..

[74] Horiuchi M., Junko E., Akatsuka S., Hasegawa T., Yamamoto E., Uno T., Kikuchi S. (2015). An effective strategy to reduce blood pressure after forest walking in middle-aged and aged people. J. Phys. Ther. Sci..

[75] Kobayashi H., Song C., Ikei H., Park B.J., Lee J., Kagawa T., Miyazaki Y. (2017). Population-based study on the effect of a forest environment on salivary cortisol concentration. Int. J. Environ. Res. Public Health.

[76] Song C., Ikei H., Miyazaki Y. (2015). Elucidation of a physiological adjustment effect in a forest environment: A pilot study. Int. J. Environ. Res. Public Health.

[77] Kim B.J., Jeong H., Park S., Lee S. (2015). Forest adjuvant anti-cancer therapy to enhance natural cytotoxicity in urban women with breast cancer: A preliminary prospective interventional study. Eur. J. Integr. Med..

[78] Barnes M.R., Donahue M.L., Keeler B.L., Shorb C.M., Mohtadi T.Z., Shelby L.J. (2019). Characterizing nature and participant experience in studies of nature exposure for positive mental health: An integrative review. Front. Psychol..

[79] An K.W., Kim E., Jeon K.S., Setsu T. (2004). Effects of forest stand density on human’s physiopsychological changes. J. Fac. Agric. Kyushu Univ..

[80] Nazaruk J., Borzym-Kluczyk M. (2015). The role of triterpenes in the management of diabetes mellitus and its complications. Phytochem. Rev..

[81] Gershenzon J., Dudareva N. (2007). The function of terpene natural products in the natural world. Nat. Chem. Biol..

[82] Mewalal R., Rai D.K., Kainer D., Chen F., Külheim C., Peter G.F., Tuskan G.A. (2017). Plant-derived terpenes: A feedstock for specialty biofuels. Trends Biotechnol..

[83] Martin D.M., Gershenzon J., Bohlmann J. (2003). Induction of volatile terpene biosynthesis and diurnal emission by methyl jasmonate in foliage of Norway spruce. Plant Physiol..

[84] Lin J.J., Lu K.W., Ma Y.S., Tang N.Y., Wu P.P., Wu C.C., Lu H.F., Lin J.G., Chung J.G. (2014). Alpha-phellandrene, a natural active monoterpene, influences a murine WEHI-3 leukemia model in vivo by enhancing macrophague phagocytosis and natural killer cell activity. In Vivo.

[85] Komori T., Fujiwara R., Tanida M., Nomura J., Yokoyama M.M. (1995). Effects of citrus fragrance on immune function and depressive states. Neuroimmunomodulation.

[86] Nam E.-S., Uhm D.-C. (2008). Effects of Phytoncides Inhalation on Serum Cortisol Level and Life Stress of College Students. Korean J. Adult Nurs..

[87] Sumitomo K., Akutsu H., Fukuyama S., Minoshima A., Kukita S., Yamamura Y., Sato Y., Hayasaka T., Osanai S., Funakoshi H. (2015). Conifer-derived monoterpenes and forest walking. Mass Spectrom..

