# Supplementary material for: How Should Forests Be Characterized in Regard to Human Health? Evidence from Existing Literature
Source: Int J Environ Res Public Health. 2020 Feb 6;17(3):1027. doi: 10.3390/ijerph17031027 (PMC7038061; doi:10.3390/ijerph17031027)
Supplement: Supplementary file 1 [file ijerph-17-01027-s001.pdf]

Table S1. Overview of the analyzed articles basic information.

Authors and reference	Year	Country	Study type	Exposure type	Exposure time	N Participants
Bang et al. [1]	2017	South Korea	Control trial	Passive	Weeks	118
Bielinis et al. [2]	2019	Poland	Before-after	Pro-active	Days	21
Chen et al. [3]	2018	Taiwan	Before-after	Pro-active	Days	16
Chun et al. [4]	2017	South Korea	Control trial	Pro-active	Days	59
Dolling et al. [5]	2017	Sweden	Comparative	Passive	Months	27
Han et al. [6]	2016	South Korea	Control trial	Pro-active	Days	61
Hassan et al. [7]	2018	China	Control trial	Passive	Minutes	60
Horiuchi et al. [8]	2013	Japan	Comparative	Passive	Hours	48
Horiuchi et al., [9]	2014	Japan	Comparative	Passive	Minutes	15
Horiuchi et al. [10]	2015	Japan	Before-after	Passive	Hours	54
Im et al. [11]	2016	South Korea	Control trial	Passive	Hours	41
Jia et al. [12]	2016	China	Control trial	Passive	Days	20
Joung et al. [13]	2015	South Korea	Control trial	Passive	Minutes	7
Kim, et al. [14]	2015	South Korea	Before-after	Passive	Days	11
Kobayashi et al. [15]	2015	Japan	Control trial	Passive	Minutes	625
Kobayashi et al. [16]	2017	Japan	Control trial	Passive	Minutes	348
Lee and Lee [17]	2014	South Korea	Control trial	Passive	Hours	43
Lee et al. [18]	2009	Japan	Control trial	Passive	Minutes	12
Lee et al. [19]	2011	Japan	Control trial	Passive	Minutes	12
Lee et al. [20]	2014	Japan	Control trial	Passive	Minutes	48
Lee et al. [21]	2018	South Korea	Comparative	Pro-active	Hours	79
Li et al. [22]	2007	Japan	Before-after	Passive	Days	12
Li et al. [23]	2008	Japan	Before-after	Passive	Days	13
Li et al. [24]	2008	Japan	Control trial	Passive	Days	12
Li et al. [25]	2011	Japan	Control trial	Passive	Days	17
Li et al. [26]	2016	Japan	Control trial	Passive	Hours	19
López-Pouza et al. [27]	2015	Spain	Comparative	Passive	Days	30
Mao et al. [28]	2012	China	Control trial	Passive	Days	20
Mao et al. [29]	2012	China	Control trial	Passive	Days	24
Mao et al. [30]	2017	China	Control trial	Passive	Days	33
Mao et al. [31]	2018	China	Control trial	Passive	Days	20
Morita et al. [32]	2011	Japan	Before-after	Passive	Hours	71
Ochiai et al. [33]	2015	Japan	Before-after	Pro-active	Hours	17

Ochiai et al. [34]	2015	Japan	Before-after	Passive	Hours	9
Ohe et al. [35]	2017	Japan	Before-after	Pro-active	Days	43
Ohtsuka et al. [36]	1998	Japan	Before-after	Passive	Years	237
Park et al. [37]	2007	Japan	Control trial	Passive	Minutes	12
Park et al. [38]	2008	Japan	Control trial	Passive	Minutes	12
Park et al. [39]	2009	Japan	Control trial	Passive	Minutes	12
Park et al. [40]	2010	Japan	Control trial	Passive	Minutes	280
Saito et al. [41]	2019	Japan	Comparative	Passive	Minutes	17
Seo et al. [42]	2015	South Korea	Before-after	Pro-active	Days	48
Shin and Choi [43]	2019	South Korea	Control trial	Passive	Minutes	10
Song et al. [44]	2013	Japan	Control trial	Passive	Minutes	485
Song et al. [45]	2015	Japan	Control trial	Passive	Minutes	20
Song et al. [46]	2015	Japan	Control trial	Passive	Minutes	92
Song et al. [47]	2017	Japan	Before-after	Pro-active	Days	26
Song et al. [48]	2017	Japan	Control trial	Passive	Minutes	20
Song et al. [49]	2019	China	Control trial	Passive	Minutes	60
Sonntag-Öström et al. [50]	2014	Sweden	Control trial	Passive	Minutes	20
Sonntag-Öström et al. [51]	2015	Sweden	Control trial	Pro-active	Days	86
Stigsdotter et al. [52]	2017	Denmark	Control trial	Passive	Minutes	51
Sung et al. [53]	2012	South Korea	Control trial	Pro-active	Days	56
Toda and Takeshita [54]	2015	Japan	Control trial	Passive	Minutes	20
Toda et al. [55]	2013	Japan	Control trial	Passive	Minutes	20
Tsao et al. [56]	2018	Taiwan	Control trial	Passive	Days	11
Tsunetsugu et al. [57]	2007	Japan	Control trial	Passive	Minutes	12
Wang et al. [58]	2018	Japan	Control trial	Passive	Hours	28
Wu et al. [59]	2017	China	Control trial	Passive	Days	33
Yamaguchi et al. [60]	2006	Japan	Control trial	Passive	Days	10
Yu et al. [61]	2016	South Korea	Before-after	Pro-active	Days	24
Yu et al. [62]	2017	Taiwan	Before-after	Pro-active	Hours	128

Table S2. Health variables registered in the articles analysis classified by systems or functions.

Endocrine/reproductive
Glucose (serum concentration)
Glycated hemoglobin (HbA1C)
Insulin
Adiponectin
Estradiol
Progesterone
Dehydroepiandrosterone sulfate (DHEA-S)
**Cardiovascular**
Diastolic blood pressure (DBP)
Systolic blood pressure (SBP)
Pulse pressure (SBP-DBP)
Cardio-ankle vascular index (CAVI)
Heart rate variability (HRV)
Mean heart rate (HR)
Standard deviation of normal to normal beat interval (SDNN)
High-frequency (HF) band
Low-frequency (LF) band
LF/HF ratio.
Homocysteine (HCY)
Renin-angiotensin system
Angiotensin II receptor type 1 (AT1)
Angiotensin II receptor type 1 (AT2)
Angiotensinogen (AGT)
Renin
Angiotensin (ANG)
Endothelin-1 (ET-1)
Brain natriuretic peptide (BNP)
Cerebral oxygenated hemoglobin (HbO2) and deoxygenated hemoglobin (HHb)
N-terminal pro-B-type natriuretic peptide (NT-proBNP) (serum concentration)
**Metabolic**
Body fat
Low-density lipoprotein (LDL) cholesterol
High-density lipoprotein (HDL) cholesterol
Remnant-like particle (RLP) cholesterol
Stress
Cortisol (serum, saliva)
Chromogranin-A (CgA) (saliva)
Adrenaline (urinary, blood)
Noradrenaline
Salivary amylase (sAMY) activity (and its natural logarithm, $\log_e$ sAMY).
Oxidative stress/carcinogenesis
Hydroperoxides (reactive oxygen metabolites of the d-ROM test)
Hydrogen peroxide (H_2O_2)
8-hydroxy-2'-deoxyguanosine (8-OHdG)
Malondialdehyde (MDA)
Superoxide dismutases (SODs) (total in serum)
Tissue inhibitor of metalloproteinase (TIMP-1) (serum concentration)
**Nervous**
Noradrenaline
Dopamine
Sleep quality assessment

Total time in bed
Sleep latency
Total sleep duration
Actual sleep
Immobile minutes
Sleep efficiency
Brain bioelectrical activity
Beta waves
Cerebral oxygenated hemoglobin (HbO_2) and deoxygenated hemoglobin (HHb)
Necker cube pattern control task (spontaneous reversals and focused reversals)
**Respiratory**
Forced vital capacity (FVC)
Forced expiratory volume in the first second (FEV1)
Forced expiratory volume in six seconds (FEV6)
Fractional exhaled nitric oxide (FeNO)
Pulmonary and activation-regulated chemokine/CC-chemokine ligand-18 (PARC/CCL18) (serum concentration)
Surfactant protein D (SP-D) (serum concentration)
**Hematological/immunological/inflammatory**
Red blood cell (RBC) count
Hemoglobin (Hb) (total)
Platelet count
White blood cell (WBC) count
CD8+ cells (proportion)
Natural killer (NK) cell count (total) and activity (proportion of activating NK cells)
NK T-like cells (proportion)
T cells (proportion)
Perforin production (total and proportion in CD8+ cells and NK cells)
Granulysin (GRN) (total and proportion)
Granzyme A and B (GrA/B) production in peripheral blood lymphocytes (PBL) and specifically in CD8+, NK and NKT-like cells (total and proportion)
Granulocytes (proportion)
Monocytes (proportion)
Macrophages (proportion)
Lymphocytes (proportion)
Interleukin-6 (IL-6)
Interleukin-8 (IL8)
Interferon-y (IFN-y)
Interleukin-1β (IL-1β)
C-reactive protein (CRP)
Tumor necrosis factor α (TNF-α)
Secretory immunoglobulin A (s-IgA) (saliva concentration)
Thymus and activation-regulated chemokine (TARC/CCL17)
Macrophage-derived chemokine (MDC/CCL22)
**Musculoskeletal**
Bone density

Supplementary references

1. Bang, K.S.; Lee, I.; Kim, S.; Lim, C.S.; Joh, H.K.; Park, B.J.; Song, M.K. The effects of a campus Forest-Walking program on undergraduate and graduate students' physical and psychological health. *Int. J. Environ. Res. Public Health* **2017**, *14*.
2. Bielinis, E.; Bielinis, L.; Krupińska-Szeluga, S.; Łukowski, A.; Takayama, N. The Effects of a Short Forest Recreation Program on Physiological and Psychological Relaxation in Young Polish Adults. *Forests* **2019**, *10*, 34.
3. Chen, H.T.; Yu, C.P.; Lee, H.Y. The effects of forest bathing on stress recovery: Evidence from middle-aged females of Taiwan. *Forests* **2018**, *8*, 1–9.
4. Chun, M.H.; Chang, M.C.; Lee, S.J. The effects of forest therapy on depression and anxiety in patients with chronic stroke. *Int. J. Neurosci.* **2017**, *127*, 199–203.
5. Dolling, A.; Nilsson, H.; Lundell, Y. Stress recovery in forest or handicraft environments – An intervention study. *Urban For. Urban Green.* **2017**, *27*, 162–172.
6. Han, J.-W.; Choi, H.; Jeon, Y.-H.; Yoon, C.-H.; Woo, J.-M.; Kim, W. The Effects of Forest Therapy on Coping with Chronic Widespread Pain: Physiological and Psychological Differences between Participants in a Forest Therapy Program and a Control Group. *Int. J. Environ. Res. Public Health* **2016**, *13*, 255.
7. Hassan, A.; Tao, J.; Li, G.; Jiang, M.; Aii, L.; Zhihui, J.; Zongfang, L.; Qibing, C. Effects of Walking in Bamboo Forest and City Environments on Brainwave Activity in Young Adults. *Evidence-Based Complement. Altern. Med.* **2018**, *2018*, 1–9.
8. Horiuchi, M.; Endo, J.; Akatsuka, S.; Uno, T.; Hasegawa, T. Influence of Forest Walking on Blood Pressure, Profile of Mood States and Stress Markers from the Viewpoint of Aging. *J. Aging Gerontol.* **2013**, *1*, 9–17.
9. Horiuchi, M.; Endo, J.; Takayama, N.; Murase, K.; Nishiyama, N.; Saito, H.; Fujiwara, A. Impact of viewing vs. Not viewing a real forest on physiological and psychological responses in the same setting. *Int. J. Environ. Res. Public Health* **2014**, *11*, 10883–10901.
10. Horiuchi, M.; Junko, E.; Akatsuka, S.; Hasegawa, T.; Yamamoto, E.; Uno, T.; Kikuchi, S. An effective strategy to reduce blood pressure after forest walking in middle-aged and aged people. *J. Phys. Ther. Sci.* **2015**, *27*, 3711–3716.
11. Im, S.G.; Choi, H.; Jeon, Y.H.; Song, M.K.; Kim, W.; Woo, J.M. Comparison of effect of two-hour exposure to forest and urban environments on cytokine, anti-oxidant, and stress levels in young adults. *Int. J. Environ. Res. Public Health* **2016**, *13*.
12. Jia, B.B.; Yang, Z.X.; Mao, G.X.; Lyu, Y.D.; Wen, X.L.; Xu, W.H.; Lyu, X.L.; Cao, Y.B.; Wang, G.F. Health Effect of Forest Bathing Trip on Elderly Patients with Chronic Obstructive Pulmonary Disease. *Biomed. Environ. Sci.* **2016**, *29*, 212–218.
13. Joung, D.; Kim, G.; Choi, Y.; Lim, H.; Park, S.; Woo, J.M.; Park, B.J. The prefrontal cortex activity and psychological effects of viewing forest landscapes in Autumn season. *Int. J. Environ. Res. Public Health* **2015**, *12*, 7235–7243.
14. Kim, B.J.; Jeong, H.; Park, S.; Lee, S. Forest adjuvant anti-cancer therapy to enhance natural cytotoxicity in urban women with breast cancer: A preliminary prospective interventional study. *Eur. J. Integr. Med.* **2015**, *7*, 474–478.
15. Kobayashi, H.; Song, C.; Ikei, H.; Kagawa, T.; Miyazaki, Y. Analysis of Individual Variations in Autonomic Responses to Urban and Forest Environments. *Evidence-Based Complement. Altern. Med.* **2015**, *2015*, 1–7.
16. Kobayashi, H.; Song, C.; Ikei, H.; Park, B.J.; Lee, J.; Kagawa, T.; Miyazaki, Y. Population-based study on the effect of a forest environment on salivary cortisol concentration. *Int. J. Environ. Res. Public Health* **2017**, *14*.
17. Lee, J.Y.; Lee, D.C. Cardiac and pulmonary benefits of forest walking versus city walking in elderly women: A randomised, controlled, open-label trial. *Eur. J. Integr. Med.* **2014**, *6*, 5–11.
18. Lee, J.; Park, B.J.; Tsunetsugu, Y.; Kagawa, T.; Miyazaki, Y. Restorative effects of viewing real forest landscapes, based on a comparison with urban landscapes. *Scand. J. For. Res.* **2009**, *24*, 227–234.
19. Lee, J.; Park, B.J.; Tsunetsugu, Y.; Ohira, T.; Kagawa, T.; Miyazaki, Y. Effect of forest bathing on physiological and psychological responses in young Japanese male subjects. *Public Health* **2011**, *125*,

93–100.
20. Lee, J.; Tsunetsugu, Y.; Takayama, N.; Park, B.-J.; Li, Q.; Song, C.; Komatsu, M.; Ikei, H.; Tyrväinen, L.; Kagawa, T.; et al. Influence of Forest Therapy on Cardiovascular Relaxation in Young Adults. *Evidence-Based Complement. Altern. Med.* **2014**, *2014*, 1–7.
21. Lee, K.J.; Hur, J.; Yang, K.S.; Lee, M.K.; Lee, S.J. Acute Biophysical Responses and Psychological Effects of Different Types of Forests in Patients With Metabolic Syndrome. *Environ. Behav.* **2018**, *50*, 298–323.
22. Li, Q.; Morimoto, K.; Nakadai, A.; Inagaki, H.; Katsumata, M.; Shimizu, T.; Hirata, Y.; Hirata, K.; Suzuki, H.; Miyazaki, T.; et al. Forest Bathing Enhances Human Natural Killer Activity and Expression of Anti-Cancer Proteins. *Int. J. Immunopathol. Pharmacol.* **2007**, *20*, 3–8.
23. Li, Q.; Morimoto, K.; Kobayashi, M.; Inagaki, H.; Katsumata, M.; Hirata, Y.; Hirata, K.; Shimizu, T.; Li, Y.J.; Wakayama, T.; et al. A forest bathing trip increases human natural killer activity and expression of anti-cancer proteins in female subjects. *J. Biol. Regul. Homeost. Agents* **2008**, *22*.
24. Li, Q.; Morimoto, K.I.; Kobayashi, M.; Inagaki, H.; Katsumata, M.; Hirata, Y.; Hirata, K.; Suzuki, H.; Li, Y.; Wakayama, Y.; et al. VISITING A FOREST, BUT NOT A CITY, INCREASES HUMAN NATURAL KILLER ACTIVITY AND EXPRESSION OF ANTI-CANCER PROTEINS. *Int. J. Immunopathol. Pharmacol.* **2008**, *21*, 117–127.
25. Li, Q.; Otsuka, T.; Kobayashi, M.; Wakayama, Y.; Inagaki, H.; Katsumata, M.; Hirata, Y.; Li, Y.; Hirata, K.; Shimizu, T.; et al. Acute effects of walking in forest environments on cardiovascular and metabolic parameters. *Eur. J. Appl. Physiol.* **2011**, *111*, 2845–2853.
26. Li, Q.; Otsuka, T.; Kobayashi, M.; Wakayama, Y.; Inagaki, H.; Katsumata, M.; Hirata, Y.; Li, Y.; Hirata, K.; Shimizu, T.; et al. Effects of forest environments on cardiovascular and metabolic parameters. *Evidence-Based Complement. Altern. Med.* **2016**, *Volume 201*, 1–7.
27. López-Pousa, S.; Bassets Pagès, G.; Monserrat-Vila, S.; de Gracia Blanco, M.; Hidalgo Colomé, J.; Garre-Olmo, J. Sense of Well-Being in Patients with Fibromyalgia: Aerobic Exercise Program in a Mature Forest—A Pilot Study. *Evidence-Based Complement. Altern. Med.* **2015**, *2015*, 1–9.
28. Mao, G.X.; Lan, X.G.; Cao, Y.B.; Chen, Z.M.; He, Z.H.; LV, Y.D.; Wang, Y.Z.; Hu, X.L.; Wang, G.F.; Yan, J. Effects of Short-Term Forest Bathing on Human Health in a Broad-Leaved Evergreen Forest in Zhejiang Province, China. *Biomed. Environ. Sci.* **2012**, *25*, 317–324.
29. Mao, G.-X.; Cao, Y.-B.; Lan, X.-G.; He, Z.-H.; Chen, Z.-M.; Wang, Y.-Z.; Hu, X.-L.; Lv, Y.-D.; Wang, G.-F.; Yan, J. Therapeutic effect of forest bathing on human hypertension in the elderly. *J. Cardiol.* **2012**, *60*, 495–502.
30. Mao, G.; Cao, Y.; Wang, B.; Wang, S.; Chen, Z.; Wang, J.; Xing, W.; Ren, X.; Lv, X.; Dong, J.; et al. The salutary influence of forest bathing on elderly patients with chronic heart failure. *Int. J. Environ. Res. Public Health* **2017**, *14*.
31. Mao, G.X.; Cao, B.Y.; Yang, Y.; Chen, Z.M.; Dong, J.H.; Chen, S.S.; Wu, Q.; Lyu, X.L.; Jia, B.B.; Yan, J.; et al. Additive Benefits of Twice Forest Bathing Trips in Elderly Patients with Chronic Heart Failure. *Biomed. Environ. Sci.* **2018**, *31*, 159+.
32. Morita, E.; Imai, M.; Okawa, M.; Miyaura, T.; Miyazaki, S. A before and after comparison of the effects of forest walking on the sleep of a community-based sample of people with sleep complaints. *Biopsychosoc. Med.* **2011**, *5*, 13.
33. Ochiai, H.; Ikei, H.; Song, C.; Kobayashi, M.; Miura, T.; Kagawa, T.; Li, Q.; Kumeda, S.; Imai, M.; Miyazaki, Y. Physiological and psychological effects of a forest therapy program on middle-aged females. *Int. J. Environ. Res. Public Health* **2015**, *12*, 15222–15232.
34. Ochiai, H.; Ikei, H.; Song, C.; Kobayashi, M.; Takamatsu, A.; Miura, T.; Kagawa, T.; Li, Q.; Kumeda, S.; Imai, M.; et al. Physiological and psychological effects of forest therapy on middle-aged males with high-normal blood pressure. *Int. J. Environ. Res. Public Health* **2015**, *12*, 2532–2542.
35. Ohe, Y.; Ikei, H.; Song, C.; Miyazaki, Y. Evaluating the relaxation effects of emerging forest-therapy tourism: A multidisciplinary approach. *Tour. Manag.* **2017**.
36. Ohtsuka, Y.; Yabunaka, N.; Takayama, S. Shinrin-yoku (forest-air bathing and walking) effectively decreases blood glucose levels in diabetic patients. *Int. J. Biometeorol.* **1998**, *41*, 125–127.
37. Park, B.J.; Tsunetsugu, Y.; Kasetani, T.; Hirano, H.; Kagawa, T.; Sato, M.; Miyazaki, Y. Physiological effects of Shinrin-yoku (taking in the atmosphere of the forest) using salivary cortisol and cerebral activity as indicators. *J. Physiol. Anthropol.* **2007**, *26*, 123–128.

38. Park, B.J.; Tsunetsugu, Y.; Ishii, H.; Furuhashi, S.; Hirano, H.; Kagawa, T.; Miyazaki, Y. Physiological effects of Shinrin-yoku (taking in the atmosphere of the forest) in a mixed forest in Shinano Town, Japan. *Scand. J. For. Res.* **2008**, *23*.
39. Park, B.J.; Tsunetsugu, Y.; Kasetani, T.; Morikawa, T.; Kagawa, T.; Miyazaki, Y. Physiological effects of forest recreation in a young conifer forest in Hinokage Town, Japan. *Silva Fenn.* **2009**, *43*, 291–301.
40. Park, B.J.; Tsunetsugu, Y.; Kasetani, T.; Kagawa, T.; Miyazaki, Y. The physiological effects of Shinrin-yoku (taking in the forest atmosphere or forest bathing): Evidence from field experiments in 24 forests across Japan. *Environ. Health Prev. Med.* **2010**, *15*, 18–26.
41. Saito, H.; Horiuchi, M.; Takayama, N.; Fujiwara, A. Effects of managed forest versus unmanaged forest on physiological restoration from a stress stimulus, and the relationship with individual traits. *J. For. Res.* **2019**, *24*, 77–85.
42. Seo, S.C.; Park, S.J.; Park, C.-W.; Yoon, W.-S.; Choung, J.T.; Yoo, Y. Clinical and immunological effects of a forest trip in children with asthma and atopic dermatitis. *Iran. J. Allergy, Asthma Immunol.* **2015**, *14*, 28–36.
43. Shin, J.-W.; Choi, J.-H. The Effects of Single Session Forest Walking on Physiological and Psychological State of Myocardial Infarction Patients. *J. People, Plants, Environ.* **2019**, *22*, 109–118.
44. Song, C.; Ikei, H.; Lee, J.; Park, B.-J.; Kagawa, T.; Miyazaki, Y. Individual differences in the physiological effects of forest therapy based on Type A and Type B behavior patterns. *J. Physiol. Anthropol.* **2013**, *32*, 14.
45. Song, C.; Ikei, H.; Kobayashi, M.; Miura, T.; Taue, M.; Kagawa, T.; Li, Q.; Kumeda, S.; Imai, M.; Miyazaki, Y. Effect of forest walking on autonomic nervous system activity in middle-aged hypertensive individuals: A pilot study. *Int. J. Environ. Res. Public Health* **2015**, *12*, 2687–2699.
46. Song, C.; Ikei, H.; Miyazaki, Y. Elucidation of a physiological adjustment effect in a forest environment: A pilot study. *Int. J. Environ. Res. Public Health* **2015**, *12*, 4247–4255.
47. Song, C.; Ikei, H.; Miyazaki, Y. Sustained effects of a forest therapy program on the blood pressure of office workers. *Urban For. Urban Green.* **2017**, *27*, 246–252.
48. Song, C.; Ikei, H.; Kobayashi, M.; Miura, T.; Li, Q.; Kagawa, T.; Kumeda, S.; Imai, M.; Miyazaki, Y. Effects of viewing forest landscape on middle-aged hypertensive men. *Urban For. Urban Green.* **2017**, *21*, 247–252.
49. Song, C.; Ikei, H.; Kagawa, T.; Miyazaki, Y. Effects of Walking in a Forest on Young Women. *Int. J. Environ. Res. Public Health* **2019**, *16*, 229.
50. Sonntag-Öström, E.; Nordin, M.; Lundell, Y.; Dolling, A.; Wiklund, U.; Karlsson, M.; Carlberg, B.; Slunga Järvholm, L. Restorative effects of visits to urban and forest environments in patients with exhaustion disorder. *Urban For. Urban Green.* **2014**, *13*, 344–354.
51. Sonntag-Öström, E.; Nordin, M.; Dolling, A.; Lundell, Y.; Nilsson, L.; Slunga Järvholm, L. Can rehabilitation in boreal forests help recovery from exhaustion disorder? The randomised clinical trial ForRest. *Scand. J. For. Res.* **2015**, *30*, 732–748.
52. Stigsdotter, U.K.; Corazon, S.S.; Sidenius, U.; Kristiansen, J.; Grahn, P. It is not all bad for the grey city – A crossover study on physiological and psychological restoration in a forest and an urban environment. *Heal. Place* **2017**, *46*, 145–154.
53. Sung, J.; Woo, J.M.; Kim, W.; Lim, S.K.; Chung, E.J. The effect of cognitive behavior therapy-based "forest therapy" program on blood pressure, salivary cortisol level, and quality of life in elderly hypertensive patients. *Clin. Exp. Hypertens.* **2012**, *34*, 1–7.
54. Toda, M.; Takeshita, T. The influence of personal patterns of behavior on the physiological effects of woodland walking. *Adv Mind Body Med* **2015**, *29*, 14–18.
55. Toda, M.; Den, R.; Hasegawa-Ohira, M.; Morimoto, K. Effects of woodland walking on salivary stress markers cortisol and chromogranin A. *Complement. Ther. Med.* **2013**, *21*, 29–34.
56. Tsao, T.-M.; Tsai, M.-J.; Hwang, J.-S.; Cheng, W.-F.; Wu, C.-F.; Chou, C.-C.; Su, T.-C. Health effects of a forest environment on natural killer cells in humans: An observational pilot study. *Oncotarget* **2018**, *9*, 16501–16511.
57. Tsunetsugu, Y.; Park, B.-J.; Ishii, H.; Hirano, H.; Kagawa, T.; Miyazaki, Y. Physiological Effects of Shinrin-yoku (Taking in the Atmosphere of the Forest) in an Old-Growth Broadleaf Forest in Yamagata Prefecture, Japan. *J. Physiol. Anthropol.* **2007**, *26*, 135–142.

58. Wang, D.H.; Yamada, A.; Miyanaga, M. Changes in urinary hydrogen peroxide and 8-hydroxy-2′-deoxyguanosine levels after a forest walk: A pilot study. *Int. J. Environ. Res. Public Health* **2018**, *15*.
59. Wu, Q.; Cao, Y.; Mao, G.; Wang, S.; Fang, Y.; Tong, Q.; Huang, Q.; Wang, B.; Yan, J.; Wang, G. Effects of forest bathing on plasma endothelin-1 in elderly patients with chronic heart failure: Implications for adjunctive therapy. *Geriatr. Gerontol. Int.* **2017**, *17*, 2627–2629.
60. Yamaguchi, M.; Deguchi, M.; Miyazaki, Y. The Effects of Exercise in Forest and Urban Environments on Sympathetic Nervous Activity of Normal Young Adults. *J. Int. Med. Res.* **2006**, *34*, 152–159.
61. Yu, Y.M.; Lee, Y.J.; Kim, J.Y.; Yoon, S.B.; Shin, C.S. Effects of forest therapy camp on quality of life and stress in postmenopausal women. *Forest Sci. Technol.* **2016**, *12*, 125–129.
62. Yu, C.P.; Lin, C.M.; Tsai, M.J.; Tsai, Y.C.; Chen, C.Y. Effects of short forest bathing program on autonomic nervous system activity and mood states in middle-aged and elderly individuals. *Int. J. Environ. Res. Public Health* **2017**, *14*.
